# Prevalence of birth defects in an Arctic Russian setting from 1973 to 2011: a register-based study

**DOI:** 10.1186/1742-4755-12-3

**Published:** 2015-01-10

**Authors:** Vitaly A Postoev, Evert Nieboer, Andrej M Grjibovski, Jon Øyvind Odland

**Affiliations:** Department of Community Medicine, UiT-The Arctic University of Norway, Tromsø, Norway; International School of Public Health, Northern State Medical University, 163061 Troickij av, 51 NSMU, ISPHA, office 2519, Arkhangelsk, Russia; Department of Biochemistry and Biomedical Sciences, McMaster University, Hamilton, ON Canada; Department of International Public Health, Norwegian Institute of Public Health, Oslo, Norway; Department of Preventive Medicine, International Kazakh-Turkish University, Turkestan, Kazakhstan

**Keywords:** Birth register, Birth defects, Prevalence, Murmansk county birth registry, Russian Federation, Arctic

## Abstract

**Background:**

Birth defects (BD) constitute an important public health issue as they are the main cause of infant death. Their prevalence in Europe for 2008–2012 was 25.6 per 1000 newborns. To date, there are no population-based studies for the Russian Federation. The aim of the present study is to estimate the prevalence of BD, its forms, and changes over time in the Russian Arctic city of Monchegorsk (Murmansk County) for the period 1973–2011.

**Methods:**

The Murmansk County Birth Register and the Kola Birth Register were the primary sources of information, covering 30448 pregnancy outcomes in Monchegorsk (Murmansk County, Russia) during the study period.

**Results:**

The total perinatal prevalence of BD was 36.1/1000 live births (LB) and stillborn (SB) (95% CI = 34.0-38.2). After exclusions of minor malformations according to the European Surveillance of Congenital Anomalies guidelines, it decreased to 26.5/1000 LB plus SB (95% CI = 24.6-28.3). The perinatal prevalence of BD that are obligatory to report in Russia was 7.3/1000 LB plus SB (95% CI = 6.4-8.3). There was a significant positive time-trend in total perinatal prevalence of birth defects across the study period (p < 0.001 for trend). Prevalence of all BD increased from 23.5/1000 to 46.3/1000 (LB plus SB), while that excluding minor defects rose from 17.7/1000 to 35.7/1000 (LB plus SB). The most prevalent group of defects was malformations of the musculoskeletal system, which represented 35.4% of all BD. The most prominent increase was observed for the urinary system, rising from 0.2/1000 to 19.1/1000 (LB plus SB).

**Conclusions:**

The observed perinatal prevalence of BD in Monchegorsk increased two-fold during the 38-year study period. Further investigations to identify the underlying bases for the observed progressive growth in BD are recommended.

## Background

Birth defects (BD) are recognized by the World Health Organization (WHO) as structural or functional anomalies that are present from birth. They represent the main causes of infant deaths and morbidity in developed countries. The prevalence of BD in Europe is reported as 25.6/1000 newborns [live births (LB) plus stillborn (SB)] for the period 2008–2012 (European Surveillance of Congenital Anomalies, EUROCAT, data [1]). About 20% of deaths under one year of age are due to congenital anomalies [2].

Birth defects are a wide group of ontogenetic disorders which can be caused by single gene defects, chromosomal disorders, multifactorial inheritance factors, occupational/environmental teratogens, micronutrient deficiencies, among other risks [3].

It has been reported that 42% of perinatal deaths are directly or indirectly connected with BD in the Russian Federation (RF) [4]. However, the estimates of BD prevalence in the RF cannot be considered comparable with those derived from European and world-based registers because of differences in surveillance protocols (e.g., the limited number of BD forms requiring mandatory registration in Russia) [5]. There is only one register in the Moscow Oblast, which joined the International Clearinghouse for Birth Defects Surveillance and Research (ICBDSR) as a member in 2001 [6]. This organization collects data for about 40 of the most severe anomalies and, based on this, the prevalence of BD in the Moscow County was reported as 12.3/1000 (LB plus SB) in 2001 and 6.1/1000 (LB plus SB) in 2009 [6, 7].

For the period March 1973 through 2002, Vaktskjold et al. reported a high prevalence (13.3/1000 of newborns) of musculoskeletal malformations in Monchegorsk [8], but not of genital defects (4.4/1000 newborns) [9]. Furthermore, for 1995–2004 Petrova and Vaktskjold found a higher incidence of neural tube defects (2.1/1000 newborns and abortions) in Arkhangelsk, Russia than in Norway [10]; by contrast, the incidences of anterior abdominal wall defects were the same (0.5/1000 newborns and abortions) [11]. Their study was based on a regional BD register.

There are 22 forms of BD (20 isolated forms, Down’s syndrome and multiple BD) for which registration is now obligatory in Russia (see Table 1 for list) [5, 12, 13]. The monitoring of BD in the RF is conducted by the Moscow Institute of Pediatrics and Children Surgery; their data contains information for 42 regional registers in addition to that for the Moscow Oblast mentioned above [12]. Based on this source, the prevalence in 2011 of all BD in RF was 23.2/1000 newborns and spanned from 7.0/1000 in Stavropol County to 50.0/1000 in Severnaya Osetia-Alanya County. By comparison, the prevalence of the mandatory BD in the RF for 2011 was 7.0/1000 newborns and ranged from 2.8/1000 newborns in Magadan County to 13.5/1000 in Ivanovo County; in Arkhangelsk County it was 10.5/1000 for all BD and 7.0/1000 for mandatory registered BD [13].

**Table 1:** **Birth defects that require mandatory reporting in the Russian Federation** [11]

Birth defects	Code by ICD-10
Anencephaly	Q00
Spina bifida	Q05
Encephalocele	Q01
Congenital hydrocephalus	Q03
Anophthalmos, microophthalmos	Q11.0, Q11.2
Anotia, microtia,	Q16.0, Q17.2
Transposition of large vessels	Q20.3
Hypoplastic left heart	Q23.4
Cleft palate	Q35
Cleft lip with or without cleft palate	Q36.0, Q36.9, Q37
Oesophageal atresia	Q39.0 – Q39.4
Ano-rectal atresia	Q42.0 – Q42.3
Hypospadias	Q54.0 – Q54.3, Q54.8, Q54.9
Renal agenesis or disgenesis	Q60.1, Q60.4, Q60.6
Epispadias	Q64.0
Urine bladder exstrophy	Q64.1
Reducing limb malformations	Q71- Q73
Diaphragmatic hernia	Q79.0
Omphalocele	Q79.2
Gastroschisis	Q79.3
Multiple congenital malformations	Q89.7
Down syndrome	Q90

**Figure 1:**
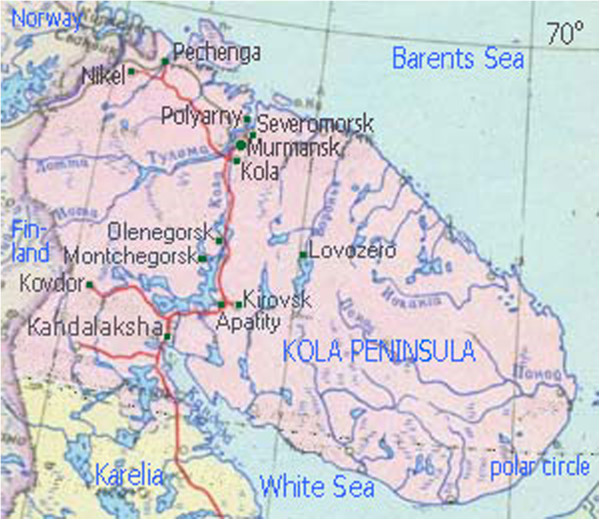
**Map of Murmansk County with surrounding areas (with permission of** http://www.briard.ru **).**

A county-wide population-based birth register established in 2005 for Murmansk County has facilitated more detailed investigation of BD epidemiology at the population level. Since living in an industrialized region of the North-west Russian Arctic might influence reproductive health and pregnancy outcomes, the city of Monchegorsk located in the Kola Peninsula was selected for an analysis of the total prevalence of BD, the types observed, and changes therein during the period 1973–2011. Monchegorsk is the one of largest cities in Murmansk County (Figure 1) with 47 403 inhabitants in 2012 [14], and its nickel refinery complex has been and remains the largest employer. The official birth rate in Monchegorsk in 2012 was 11.3/1000 and resembled the regional birth rate (11.8/1000); perinatal mortality rates were also comparable (respectively, 7.47/1000 and 7.22/1000) [14]. Similarly, for all of Russia the 2012 rates were 13.3/1000 (of births) and 8.6/1000 (perinatal mortality) [15].

The aim of the study was to estimate the prevalence and structure of BD during 1973–2011 in Monchegorsk, Northwest Russia, using established Russian birth registers and EUROCAT guidelines.

## Methods

### Population and sources of information

The study population included all newborns delivered in Monchegorsk and registered in either the Kola Birth Register (KBR) or the Murmansk County Birth Register (MCBR) for the years 1973–2011.

The KBR was established in 1998 by the retrospective collection of information about all births from week 28 of gestation on that occurred in Monchegorsk as of March 1973. Registration was continued prospectively until 2005. In addition, 102 newborns with age 13–27 weeks of gestation were also registered, of which 82 were stillborn or died during the first week of life. Details of the construction and description of the register’s suitability for epidemiological investigations have been published [8, 9, 16]. A total number of 26 841 pregnancy outcomes were registered. The implementation and use of the KBR demonstrated the need for a medical birth registry for the entire Murmansk region (also referred to as the Kola Peninsula).

The MCBR was established in 2005, and the prospective registration of pregnancy outcomes from 22 weeks of gestation began on the 1^st^ of January 2006 [17]. A total of 3750 births in Monchegorsk were registered during 2006–2011.

Data from the two registers were merged into one database using the same fields: maternal date of birth; child’s birth date; and status of child (LB or SB); BD diagnosis and its ICD-10 code. Newborns were excluded from the study if diagnoses at birth had been missed, or its interpretation had been uncertain (i.e., not having an ICD-10 code). We also excluded newborns with missing data about their status at birth. The total number of excluded newborns was 144 (0.5%), and thus 30448 newborns were included in the current analysis.

The registration of BD in the two registers included information about the existence of BD and its diagnosis; the latter was based on primary medical documentation (up to five fields of diagnosis), and were coded according to ICD-10. The diagnosis was usually made by a pediatrician or neonatologist after birth and during the maternity house stay. Use of pertinent diagnostic tools such as ultrasound examination (US) or echocardiography supplemented routine examinations. In some cases, prenatal US evidence for suspected BD was confirmed by examination after birth. In case of abortion or fetal death, the diagnosis was based on autopsy results.

### Data analysis

The prevalence, structure of BD, and distribution of the different forms were estimated based on the two- and three-level of International Classification of Disease, the Tenth Edition (ICD-10), Chapter XVII, Q codes. The newborns with more than one BD diagnosis were included in the analysis only as newborns with multiple defects as their diagnoses were not coded as Q89.7 (“multiple congenital malformations, not elsewhere classified”). These newborns were not included for any specific defects they had.

The total prevalence and prevalence by BD groups were calculated using the number of newborns (LB plus SB) with BD as the numerator, with the total number of newborns included as the denominator. To make the prevalence data more comparable with those of EUROCAT, we calculated the total BD prevalence and that of all BD malformation groups after excluding all minor anomalies according to the EUROCAT Guide 1.4, Chapter 3.2: “Minor malformation for exclusion” from the total number of BD [18]. The prevalence of malformations for which registration was mandatory in the RF was calculated using the sum of newborns with the BD items listed in Table 1 as the numerator.

Descriptive statistics were calculated using SPSS 21.0. The rates are presented per 1000 newborns (LB plus SB), with 95% confidence intervals (95% CI). A time-trend calculation was done for four 10-years periods (the last one included nine years) using chi-squared test for trend.

### Ethical considerations

The Committee for Research Ethics at the Northern State Medical University (Arkhangelsk, Russia) and REK Regional Committee for Health and Research Ethics, Northern Norway (Tromsø, Norway) approved the current study.

## Results

There were 1099 newborns with BD in Monchegorsk during the 1973–2011 study period. The total prevalence was 36.1/1000 newborns (95% CI = 34.0-38.2). There were 96 cases (8.7%) of multiple BD [3.2/1000 (95% CI = 2.5-3.8)] among them. The prevalence among LB was 34.7/1000 (95% CI = 32.6-36.9), and 167.3/1000 for SB (95% CI = 123.4-211.2). The most prevalent defect groups were congenital malformations and deformations of the musculoskeletal system, which represented 35.4% of all birth defects (i.e., 386 cases).

After exclusion of minor anomalies, 808 cases of BD among 30448 newborn were identified and resulted in a prevalence rate of 26.5/1000 (95% CI = 24.6-28.3). Those for all BD groups were calculated for both the total number of BD and after the exclusion of minor BD cases; they are presented in Table 2. Comparable data for the 2008–2012 period derived from the EUROCAT Prevalence Tables [1] are presented in Table 3.

**Table 2:** **The perinatal prevalence of birth defects grouped according to ICD-10**

Group of birth defect (ICD-10 codes)	Total prevalence ^1^		Prevalence, excl. minor anomalies ^2^	
	Prevalence	95% CI	Prevalence	95% CI
Congenital malformations of the nervous system (Q00-Q07)	1.8	1.3-2.2	1.8	1.3-2.2
Congenital malformations of eye, ear, face and neck (Q10-Q18)	0.6	0.3-0.8	0.4	0.2-0.7
Congenital malformations of the circulatory system (Q20-Q28)	2.1	1.6-2.7	2.1	1.6-2.6
Congenital malformations of the respiratory system(Q30-Q34)	1.0	0.6-1.4	0.5	0.2-0.7
Cleft lip and cleft palate (Q35-Q37)	1.2	0.8-1.6	1.2	0.8-1.6
Other congenital malformations of the digestive system (Q38-Q45)	1.2	0.8-1.6	0.8	0.5-1.1
Congenital malformations of genital organs (Q50-Q56)	3.7	3.0-4.4	2.1	1.6-2.6
Congenital malformations of the urinary system (Q60-Q64)	4.4	3.7-5.1	4.3	3.6-5.1
Congenital malformations and deformations of the musculoskeletal system(Q65-Q79)	12.7	11.4-13.9	8.7	7.7-9.7
Other congenital malformations, excluding multiple (Q80-Q89, excluding Q 89.7)	3.6	2.9-4.3	0.9	0.6-1.0
Chromosomal abnormalities, not elsewhere classified (Q90-Q99)	0.7	0.4-1.1	0.7	0.4-1.0
Multiple congenital malformation, not classified	3.2	2.5-3.8	3.0	2.4-3.6

^1^All BD, including minor defects; per 1000 newborns (LB plus SB).

^2^All minor anomalies were excluded according to EUROCAT guidelines; per 1000 newborns (LB plus SB).

**Table 3:** **Comparison of BD prevalences in Monchegorsk for 2003–2011 (excluding minor defects as per EUROCAT guidelines) with EUROCAT data for Europe** [1]

Group of birth defect	Monchegorsk 2003-2011 ^1,2^	EUROCAT 2008-2012 ^2^
Congenital malformations of the nervous system	1.9 (0.7-3.0)	2.5 (2.5-2.6)
Congenital malformations of eye, ear, face and neck	0.7 (0–1.5)	0.6 (0.55- 0.65)
Congenital malformations of the circulatory system	1.1 (0.2-2.0)	8.0 (7.9-8.1)
Congenital malformations of the respiratory system	0.4 (0–0.9)	0.7 (0.65-0.75)
Cleft lip and cleft palate	1.1 (0.2-2.0)	1.4 (1.3-1.5)
Other congenital malformations of the digestive system	0.9 (0.1-1.7)	1.8 (1.7-1.9)
Congenital malformations of genital organs	3.1 (1.7-4.6)	2.2 (2.1-2.2)
Congenital malformations of the urinary system	19.1 (15.4-22.7)	3.3 (3.3 – 3.4)
Congenital malformations and deformations of the musculoskeletal system	4.6 (2.8-6.4)	4.1 (4.0-4.2)
Other congenital malformations, excluding multiple	1.3 (0.3-2.3)	1.2 (1.1–1.3)
Chromosomal abnormalities, not elsewhere classified	0.7 (0–1.5)	3.9 (3.8–4.0)

^1^Prevalence per 1000 newborns (LB plus SB), with 95% CI in parentheses.

^2^Newborns with multiple malformations were not included in the analysis.

The prevalence of BD (LB + SB) for which reporting was mandatory was 7.3/1000 newborns (95% CI = 6.4-8.3). For LB it was 6.8/1000 (95% CI = 5.8-7.7) and 67.2/1000 (95% CI = 38.1-97.2) for SB. The prevalence rates stratified by specific defects (LB + SB) are summarized in Table 4.

**Table 4:** **Perinatal prevalence of birth defects in Monchegorsk that require mandatory reporting in the Russian Federation**

Birth defects	Total number in Monchegorsk	Prevalence in Monchegorsk ^1^
Anencephaly	7	0.2 (0.1-0.4)
Spina bifida	17	0.6 (0.3-0.8)
Encephalocele	0	0
Congenital hydrocephalus	31	1.0 (0.7-1.4)
Anophthalmos, microophthalmos	2	0.1 (0–0.2)
Anotia, microtia,	2	0.1 (0–0.2)
Transposition of large vessels	1	0.03 (0–0.1)
Hypoplastic left heart	0	0
Cleft palate	16	0.5 (0.3-0.8)
Cleft lip with or without cleft palate	27	0.9 (0.6-1.2)
Oesophageal atresia	6	0.2 (0–0.4)
Ano-rectal atresia	3	0.1 (0–0.2)
Hypospadias	53	1.7 (1.3-2.2)
Renal agenesis or disgenesis	2	0.1 (0–0.2)
Epispadias	1	0.03 (0–0.1)
Urine bladder exstrophy	3	0.1 (0–0.2)
Reducing limb malformations	13	0.4 (0.2-0.7)
Diaphragmatic hernia	1	0.03 (0–0.1)
Omphalocele	4	0.1 (0–0.3)
Gastroschisis	2	0.1 (0–0.2)
Multiple congenital	2	0.1 (0–0.2)
Down Syndrome	30	1.0 (0.6-1.3)
Total rate of BD mandatory for registration	223	7.3 (6.4-8.3)

^1^Prevalence per 1000 newborns (LB plus SB) and 95% CI.

**Figure 2:**
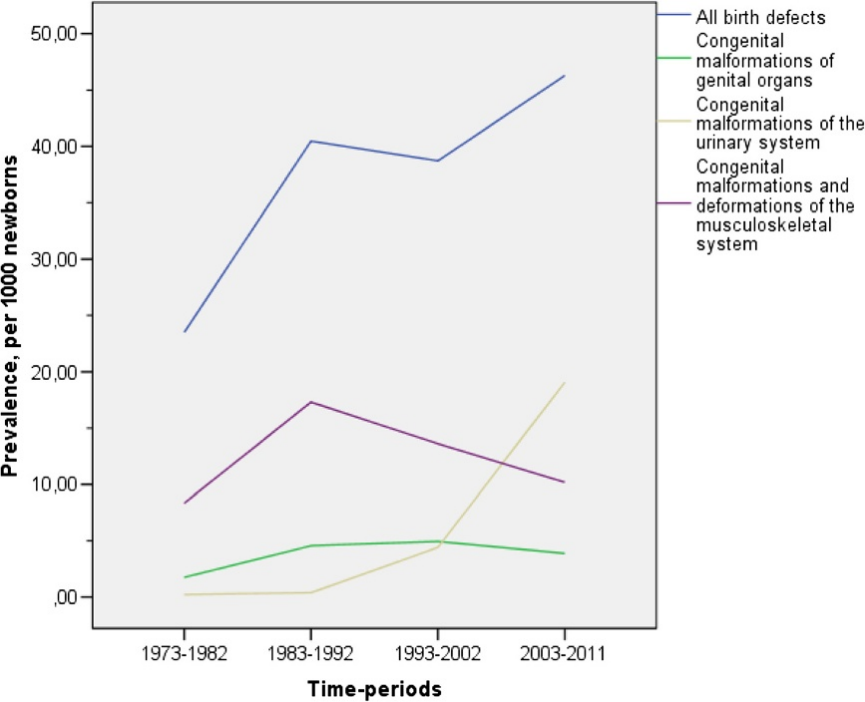
**Prevalence of all birth defects and the most prevalent groups of defects in Monchegorsk, stratified by calendar periods.**

A significant positive time-trend for the total BD prevalence among newborns was observed, as well as for malformations of the nervous system, those involving 'eye, ear, face and neck’, the genital organs, and of the urinary system. Changes in BD prevalence across the four time-periods are summarized in Tables 5 and 6 and depicted in Figure 2. After exclusion of urinary system malformations, which exceeded 40% of the total in 2003–2011, the overall prevalence was 31.8/1000 (95% CI = 29.9-33.8) and the observed time trend became non-significant. In this instance, the prevalence of BD changed from 22.3/1000 (95% CI = 20.2-26.4) in 1973–1982 to 27.8/1000 (95% CI = 23.3-32.2) in 2003–2011 with a peak of 40.1/1000 (95% CI = 36.3-43.9) in 1983–1992.

**Table 5:** **Prevalence of BD in Monchegorsk, stratified by time-periods**

Prevalence ^1^	73-82	83-92	93-02	03-11	p-value for trend
Total perinatal prevalence	23.5	40.5	38.7	46.3	<0.0001
	(20.4-26.6)	(36.7-44.3)	(33.7-43.7)	(40.7-51.9)	
Prevalence by EUROCAT guideline	17.7	26.0	31.1	37.8	<0.0001
	(15.0-20.4)	(22.9-29.1)	(26.6-35.6)	(32.7-42.9)	
Mandatory BD (22 forms)	4.8	7.6	11.3	6.9	0.11
	(3.4-6.3)	(5.9-9.3)	(8.6-14.1)	(4.7-9.1)	

^1^Per 1000 newborns (LB plus SB) with the 95% CI presented in parentheses.

**Table 6:** **Prevalence** ^**1**^ **of groups of BD in Monchegorsk, stratified by time-period**

Group of birth defect	73-82	83-92	93-02	03-11	p-value for trend
Congenital malformations of the nervous system	0.8	2.0	2.8	1.9	0.04
	(0.2-1.3)	(1.2-2.9)	(1.4-4.2)	(0.7-3.9)	
Congenital malformations of eye, ear, face and neck	0.2	0.3	1.1	1.1	0.02
	(0–0.5)	(0–0.6)	(0.2-1.9)	(0.2-2.0)	
Congenital malformations of the circulatory system	2.4	1.9	2.8	1.3	0.37
	(1.4-3.4)	(1.1-2.8)	(1.4-4.2)	(0.3-2.3)	
Congenital malformations of the respiratory system	1.3	0.6	1.4	0.6	0.38
	(0.6-2.1)	(0.1-1.1)	(0.4-2.4)	(0–1.2)	
Cleft lip and cleft palate	1.3	0.7	2.1	1.9	0.46
	(0.6-2.1)	(0.2-1.2)	(0.9-3.3)	(0.3-2.3)	
Other congenital malformations of the digestive system	1.4	1.3	0.7	1.1	0.37
	(0.7-2.2)	(0.6-2.0)	(0–1.4)	(0.2-2.0)	
Congenital malformations of genital organs	1.8	4.6	4.9	3.9	0.02
	(0.9-2.6)	(3.3-5.9)	(3.1-6.8)	(2.2-5.6)	
Congenital malformations of the urinary system	0.2	0.4	4.4	19.1	<0.0001
	(0–0.5)	(0–0.8)	(2.7-6.1)	(15.4-22.7)	
Congenital malformations and deformations of the musculoskeletal system	8.3	17.3	13.6	10.2	0.39
	(6.5-10.2)	(14.8-19.8)	(10.6-16.6)	(7.5-12.9)	
Other congenital malformations, excluding multiple	2.4	6.6	1.4	2.2	0.14
	(1.4-3.4)	(5.0-8.2)	(0.4-2.4)	(1.0-3.5)	
Chromosomal abnormalities, not elsewhere classified	1.0	0.6	0.5	0.7	0.51
	(0.3-1.6)	(0.1-1.1)	(0–1.1)	(0–1.5)	
Multiple congenital malformation, not classified	2.3	4.2	2.8	3.0	0.71
	(1.3-3.3)	(2.9-5.4)	(1.4-4.2)	(1.5-4.4)	

^1^Newborns with all BD were included in the analysis; per 1000 newborns (LB plus SB), with 95% CI in brackets.

## Discussion

To define the true incidence of BD is problematic because of the difficulty of defining the population at risk, and an inability to take into account the occurrence of unknown fetal deaths and of unknown pregnancies. Consequently, prevalence is the recommended measure [19]. In addition, Mason et al. [19] recommended that the number of stillborn not be included in the denominator, but acknowledged that not doing so “has relatively little impact on the final prevalence estimate”. We included them to make our results more comparable with the EUROCAT data. The number of fetal deaths was indeed relatively small (less than 1%), and thus our findings concur with the above statement.

### Comparisons of findings with those from other studies/registers

Although the observed prevalence of all BD for the study period was higher than reported in other European registers [1, 20], improved agreement with the latest EUROCAT data was evident after minor anomalies were excluded. However, that for the last decade was higher than in Europe, even after adjustments using the EUROCAT guidelines.

A comparison of our results with other available Russian monitoring data shows that in Monchegorsk the total prevalence was also higher than the mean Russian value for the 2003–2011 period. By contrast, the prevalences for BD that require mandatory reporting were approximately equal [13]. However, use of official monitoring data in the assessment of BD prevalences likely involve less rigorous data collection than that exercised for birth registers, and perhaps is also prone to under-reporting less severe forms of defects. Our prevalence estimates of genital malformations and defects of musculoskeletal system were lower than those reported for Monchegorsk by Vaktskjold et al. [8, 9]. Our neural tube and anterior abdominal wall defect prevalences were also lower than those reported for Arkhangelsk by Petrova and Vaktskjold as incidences (abortions after 12 weeks of gestation were included) [10, 11].

Compared to EUROCAT data (1980–2011) [1, 20], the prevalence of cardiovascular malformations in Monchegorsk was lower; those of musculoskeletal and urinary BD were higher; and of comparable magnitude for other malformations (see Table 3). Prevalences of the severe malformations for which registration in Russia is mandatory were mostly similar, but were higher for Down syndrome, severe cardiovascular malformations and diaphragmatic hernia. A likely reason for this is that EUROCAT includes cases of termination of pregnancy due to fetal anomaly (TOPFA), while in the RF mandatory autopsies of aborted fetuses are performed only after 22 weeks of gestation.

The low rate of cardiovascular defects in Monchegorsk can at least be partly explained by an underestimation of the true number of minor chamber defects because of an absence of symptoms during the first week of life. A prenatal diagnosis in regional districts of severe malformation that potentially could be surgically corrected may have led to a transfer of a delivery to regional (Murmansk) or federal (Moscow) centers. Such births were not registered in either of the registers.

Our data for BD that required mandatory reporting are comparable with those available from the Medical Birth Registry of Norway [21] for the same time frame. However, the following prevalences were higher in Norway: transposition of great vessels (0.21/1000), cleft lip with or without cleft palate (1.36/1000), Down Syndrome (1.34/1000), and lower for congenital hydrocephalus (0.43/1000) [21]. The total prevalence of all defects and deformations in the observation period years was also comparable, although the Norwegian data includes TOPFAs from 2000 and based on this we might expect lower values if they had not been.

### Interpretation of time trends

The positive trend of the total BD prevalence across the study period could be the result of an increase in the prevalence of congenital malformations of the genital organs and urinary system. However, the interpretation of such dependence is complex and might well reflect changes in the health-care system, birth registration protocols, the socio-economic situation and changes in coding practices (the KBR was established retrospectively and historical codes were re-assigned during the past 40 years to conform with ICD-10). Furthermore, the prevalence of compatible-with-living defects, such as urinary malformations, has increased because most of them can be easily visualized by US-screening [22]. Interestingly, the most severe BD that are often incompatible with life (such as anencephaly) did not exhibit substantial changes in prevalence after the 1970–1980 period. This observation presumably reflects an impact of prenatal diagnostics established in 1994, which included one US examination and more complex prenatal screening procedures after the year 2000. Evidently, these improvements in the prenatal detection led to the reduction of severe malformations and better diagnosis of minor ones at the first week of life.

An increase in the prevalence of urinary system malformations during 2003-2011was no doubt influenced by an increase of two defects, namely Q62.0 (congenital hydronephrosis) and Q63.0 (another malformations of kidney, unspecified). Both forms could be symptomless during the first days of life and thus could only have been diagnosed during the time-frame when US-examinations were conducted. On the other hand, the observed increased occurrence of these forms could also reflect over diagnosis, but this would require a detailed follow-up study to verify.

Increased prevalence of BD over time could also represent a true rise, perhaps due to environmental factors. For example, the prevalence of some group of BD (malformations of genital organs, musculoskeletal abnormalities and multiple BD) rose two- to three-fold between 1973 and 1992, which overlaps increased production at the local nickel refinery complex that reached a maximum between 1982 and 1988. The observed peak in BD prevalence during 1983–1992 (after exclusion of urinary system defects) also coincides. The primary pollutants were sulfur dioxide and particulates containing a suite of inorganic elements including toxic metals and nonmetals [23]. The latter can accumulate in soil and be transferred to the watershed over time. Although these emissions have been systematically reduced since the early 1990s, they remain substantial. Other possible explanations of increasing BD prevalence include higher maternal age and an increase in alcohol abuse and smoking among mothers; specifically, 30.8% of women in Russia of reproductive age were smokers in 2008–2010 [24] compared to 19% in 1990 [25].

### Study strengthens and limitations

Our study is the first to examine all types BD prevalences recorded in two population-based registers in Russia that can be compared with European registers. A possible limitation of our study pertains to the retrospectively established KBR database. Only diagnoses made in the maternity houses were taken into account, which could be a reason for underestimation. Minor anomalies or defects that might be revealed later (e.g., small septal heart defects without heart failure) were not identified. Neither were TOPFAs included in either database, which could mean that the prevalence could be higher than calculated after the year 2000 when prenatal screening was established. On the other hand and in terms of the register data, only a small number of newborns had missing information (0.5%). Difference in the gestational age limits of registration of the newborns in the merged registries and retrospective type of data collection in the KBR could potentially have influenced the results, although this would not explain the total BD prevalence increase seen for 2003–2011. There were 17 newborns under 28 weeks in 2006–2011 registered in the MCBR. Among them, only three additional cases of BD were found and the total number of newborns with BD for that period was 134. Inclusion of these data did not change the BD prevalence significantly. Furthermore, when the 102 newborns with gestational age less than 28 weeks noted in the KBR were included in the analysis, there was also little impact on our findings. One might have expected the prevalence of the most severe BD to have increased as well, but we did not observe this.

## Conclusion

The perinatal prevalence of BD in Monchegorsk increased during the study period and was 36.1 per 1000 newborns overall. The most prevalent groups of defects were musculoskeletal and urinary malformations. Investigation of the possible reasons for these findings is recommended.

## Abbreviations

BD: Birth defect
EUROCAT: European surveillance of congenital anomalies
ICBDSR: International clearinghouse for birth defects surveillance and research
ICD-10: the International statistical classification of diseases and related health problems, tenth revision
KBR: Kola birth register
LB: Live birth
MCBR: Murmansk County birth register
RF: Russian Federation
SB: Stillborn
TOPFA: Termination of pregnancy due to fetal anomaly
US: Ultrasound
WHO: World Health Organization.
